# Gut Microbiota and Colon Cancer: A Role for Bacterial Protein Toxins?

**DOI:** 10.3390/ijms21176201

**Published:** 2020-08-27

**Authors:** Carla Fiorentini, Francesca Carlini, Elena Angela Pia Germinario, Zaira Maroccia, Sara Travaglione, Alessia Fabbri

**Affiliations:** 1 Association for Research on Integrative Oncology Therapies (ARTOI), Via Ludovico Micara, 73, 00165 Rome, Italy; carla.fiorentini@artoi.it; 2 Department of Cardiovascular, Endocrine-Metabolic Diseases and Aging, Istituto Superiore di Sanità, Viale Regina Elena, 299, 00161 Rome, Italy; francesca.carlini@iss.it (F.C.); elena.germinario@iss.it (E.A.P.G.); zaira.maroccia@iss.it (Z.M.); sara.travaglione@iss.it (S.T.)

**Keywords:** colorectal neoplasms, gut microbiota, bacterial protein toxin, bacterial infections, inflammation, carcinogenesis, cell proliferation, DNA damage

## Abstract

Accumulating evidence indicates that the human intestinal microbiota can contribute to the etiology of colorectal cancer. Triggering factors, including inflammation and bacterial infections, may favor the shift of the gut microbiota from a mutualistic to a pro-carcinogenic configuration. In this context, certain bacterial pathogens can exert a pro-tumoral activity by producing enzymatically-active protein toxins that either directly induce host cell DNA damage or interfere with essential host cell signaling pathways involved in cell proliferation, apoptosis, and inflammation. This review is focused on those toxins that, by mimicking carcinogens and cancer promoters, could represent a paradigm for bacterially induced carcinogenesis.

## 1. Introduction

**Figure 1:**
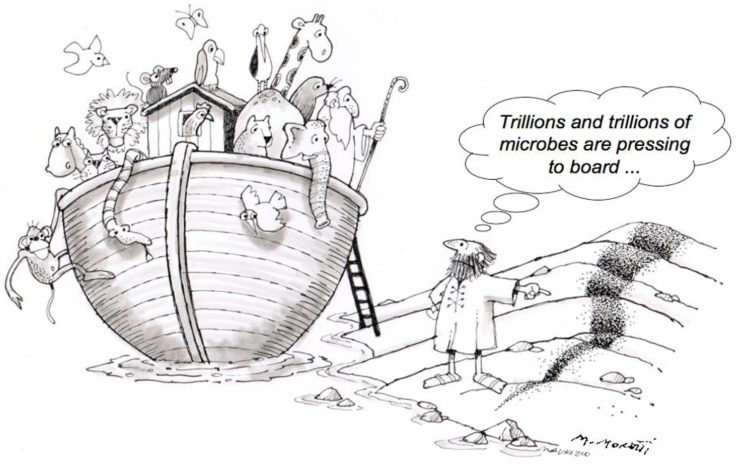
The everlasting relationship between microbes and their host. An interpretative artwork by the artist Maurizio Moretti.

Nowadays, the human body is worldwide recognized as the home to a complex community, crowded and various, composed by at least 100 trillion microbial cells [1] that have certainly coexisted with their host since the most ancient times [2] (Figure 1). The microbial ecosystem that resides in and on the human body constitutes, collectively, the microbiota, and the genes encoded are known as the microbiome. The constituents of the microbiota—bacteria, eukaryotes, viruses, and archaeon—interact with one another and with the host, significantly influencing human well-being [3]. The gut microbiota, in particular, where the great majority of microbes reside, plays a pivotal role in the control of our physical and emotional health state [4,5]. Besides, the gut microbiota may provide invaluable information in several fields, including certain aspects of our ancient past. Just out of curiosity, the microbiota discovered in the stomach of the glacier mummy Ötzi, found in the Alps, discloses important features not only on life, health, and death but also on the human migration in Europe during the Copper Age. Specifically, the *Helicobacter pylori* genome from the stomach and gastrointestinal tract of Ötzi is a nearly pure representative of the bacterial population of Asian origin that existed in Europe, hypothesizing a more recent date for hybridization between the Asian and African population [6,7,8,9,10]. The identification of the specific bacterial strain allowed to reconstruct the chronology of the bacterium’s evolution, which is intertwined with migrations and crossings between human populations in the last thousands of years. Moreover, the gut microbiota from the Hadza, a community of Tanzanian hunter–gatherers whose lifestyle closely resembles that of Paleolithic humans, may furnish interesting clues on the co-evolution of microbiota–human host. Recent studies focused on this hunter–gatherer group have revealed higher levels of microbial richness and biodiversity than urban controls [11], and an annual cyclic reconfiguration of the intestinal microbiome [12]. The microbiota changes that have taken place in industrialized urban populations may have influenced the microbiota–host co-metabolic network, thus possibly contributing to the increasing list of Western diseases.

**Figure 2:**
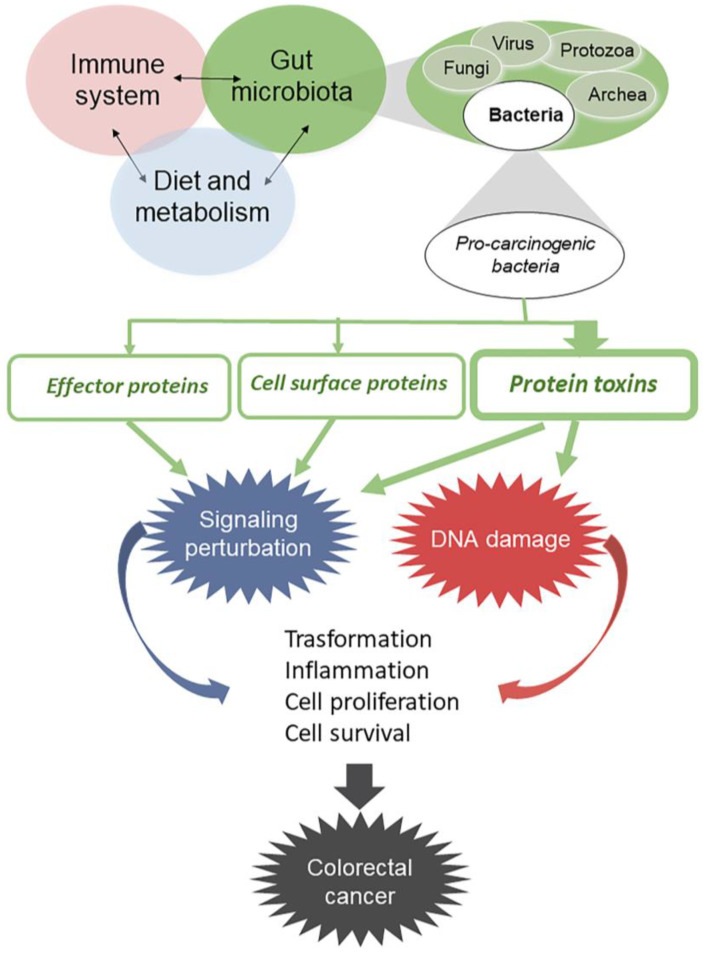
Role of bacterial products in colorectal cancer (CRC). Dysregulation of gut microbial balance, in association with inflammatory and environmental events, is recognized as one of the risk factors for CRC. As a side effect, protein toxins produced by pathogenic bacteria can have a pro-carcinogenic activity through the direct attack to DNA, with consequent genomic instability, or through alterations of cell signaling, that stimulate proliferation and induction of cell death resistance. Effectors proteins and bacterial cell surface proteins are also important pro-carcinogenic virulence factors for CRC development. Different types of arrows are used for protein toxin and effector proteins/cell surface proteins.

A huge number of studies has highlighted that the gut microbiota performs several vital functions, including the development of the immune system [13,14], metabolization of dietary compounds, and protection against the invasion and growth of gut pathogens [15,16,17]. All these pivotal metabolic functions are strongly dependent on the gut microbial balance, whose dysregulation may lead to a condition called dysbiosis that is connected to a number of human pathologies, including cancer [18]. It is interesting to note that, from a historical point of view, the involvement of the gut microbiota in the insurgence of colorectal cancer (CRC) [18] has been somehow foreseen by Hippocrates (about 400 B.C.) who has been quoted as saying “death sits in the bowels” and “bad digestion is the root of all evil” [19,20]. Hence, an altered composition of the gut microbiota, in association with inflammatory and environmental events, is recognized as one of the risk factors for CRC (Figure 2).

Bacteria represent the larger microbial portion of the gut microbiota, and several bacterial taxa contain protein toxin-producing strains that may have a pro-carcinogenic potential [21]. Information is progressively emerging on the long-term consequences of chronic exposure to such intestinal bacteria as well as the possible contribution to transformation of their toxins, although this last aspect remains poorly investigated. Protein toxins, among the principal virulence factors of pathogenic bacteria, favor their colonization and spread in the host, thus perturbing the host equilibrium and possibly causing disease. They can have, as a side effect, a pro-carcinogenic activity that may occur in various ways, including a direct attack to DNA with consequent genomic instability, or alterations of cell signaling that stimulate proliferation and the induction of cell death resistance [22]. Effector proteins and bacterial cell surface proteins are also important pro-carcinogenic virulence factors for CRC development (Figure 2).

In this review, besides updating our previous review on the same topic [23], we will discuss how the encounter of protein virulence factors produced by gut bacteria with host cells may result in the manipulation of crucial host cell pathways involved in cancer onset.

## 2. Bacterial Protein Toxins Causing Genome Instability

**Figure 3:**
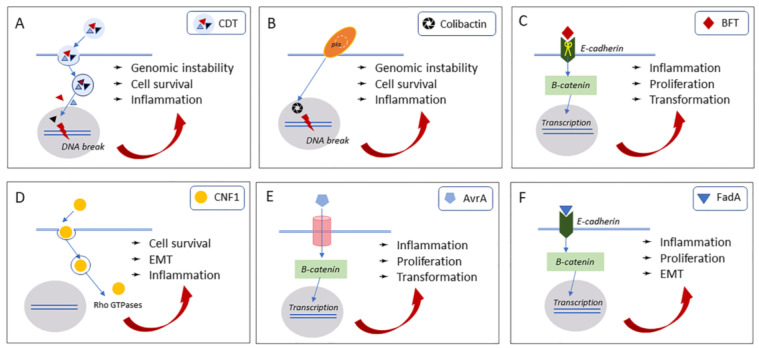
Toxic mechanisms by which virulence factors may contribute to CRC. Schematic representation of the toxic mechanism of each virulence factor described in this review. (**A**,**B**) represent Cdt and colibactin, respectively, whose mode of action contribute to CRC development or progression through genomic instability. (**C**–**F**) represent virulence factors that contribute to CRC through alteration of crucial cell signaling pathways involved in cell proliferation and cell death. It is worth noting that inflammation is somehow involved in the activity of all the herein described virulence factors. Thin blue arrows indicate the entry of the bacterial factors; thick red arrows indicate the CRC-related effects.

Genome instability is most readily caused by bacterial protein toxins that trigger host cell double-strand DNA breaks (DSBs), such as the cytolethal distending toxin (Cdt) and the colibactin (Figure 3A,B).

### 2.1. Cytolethal Distending Toxin (Cdt)

The Cdts are a group of heat-labile protein exotoxins secreted by more than 30 pathogenic Gram-negative bacteria [24], including *Salmonella typhi*, that infect mucocutaneus tissue and induce inflammatory disease [24,25,26,27,28,29,30,31,32]. Cdts are cyclomodulins that interfere with the eukaryotic cell cycle, inhibiting or interfering with the normal course of the cell cycle division, and that can modulate the host microenvironment and the host immune response (Figure 3A).

They are heterotrimers composed of two subunits, CdtA and CdtC, responsible for the binding to the host cell membrane, and of one active subunit, CdtB, which possesses enzymatic activity [33]. After cell entry by endocytosis, CdtB undergoes retrograde transport via the endosomes and Golgi to the endoplasmic reticulum (ER), where it is translocated into the cytosol through a mechanism involving ER-associated protein degradation. From the cytosol, CdtB is imported into the nucleus where it exerts its cytotoxic effects [34,35,36]. The active subunit CdtB alone is necessary and sufficient to account for cellular toxicity [36,37], having a dual DNase and phosphatase activity. CdtB induces, in host cells, DNA single-strand breaks (SSBs) at a low dose, and DSBs at high dose, with subsequent activation of the DNA Damage Response (DDR) [38,39]. DDR has the function to preserve DNA integrity and is a highly conserved danger sensing mechanism [40,41] that depends on the activation of the DNA damage checkpoint pathways. Three related kinases, the DNA-dependent protein kinase (DNA-PK), the Ataxia Telangiectasia Mutated (ATM), and the ATM-and Rad3 related (ATR) sense the damaged DNA and coordinate cell cycle arrest and repair [41]. DNA-PK and ATM have a major role in repairing DDBs, by activation of the checkpoint responses via phosphorylation of the downstream effectors CHK2 and p53 that induce cell cycle arrest. ATR pathway is mainly involved in repairing SSBs and other types of DNA lesions by activation of checkpoint responses through CHK1 phosphorylation, and DNA repair [40,41,42]. Different cell lines respond in different ways to Cdt stimuli. While epithelial, endothelial, and fibroblast cell lines undergo arrest of cell cycle progression resulting in nuclear and cytoplasmic distension that precedes apoptotic cell death, cells of hematopoietic origin follow a brief period of cell cycle arrest [43]. Failure in DNA repairing will lead to cell death by either apoptosis or senescence, depending also on the activation of the mitogen-activated protein kinase (MAPK) p38. The presence of DNA damage can stimulate the host immune response, resulting in either pro-inflammatory or anti-inflammatory reactions. Cdt has shown to influence the process of pro-inflammation inducing the production of pro-inflammatory cytokines (IL-1β, TNFα, IL-6, and IL-8) as demonstrated in several studies [44,45,46,47,48,49]. Interestingly, Cdts also possess anti-inflammatory properties, causing a decreased phagocytosis ability in macrophages, with the final result for Cdt-producing bacteria to better colonize host-tissue [48,50,51]. Prolonged exposure to sublethal doses of Cdt can impair DDRs, resulting in impaired ability in DNA damage detection and the accumulation of mutations. At the same time, MAPK activity is upregulated by activation of the neuroepithelial cell-transforming gene 1 protein and the GTPase RhoA, which supports the survival of the toxin-exposed cells. The consequence is the acquisition of the capacity to propagate mutations in DNA arising during the repair process, thus inducing genomic errors that underlie cancer formation [52]. Comparative in vitro studies have been conducted on normal human colon epithelial cell lines (HCECs) in order to state the Cdt activity on three major genes involved in CRC genetic models: Adenomatous polyposis coli (*APC*), *TP53* (p53), and Kirsten-ras (*KRAS*). The results demonstrate that *APC* and *TP53* deficient cells show an impaired DNA damage response after Cdt exposure, whereas HCECs expressing oncogenic *KRASV12* were more resistant to Cdt [53]. Although the in vivo pathogenic potential of Cdts in bacterial infection is not fully appreciated, some studies have reported the ability of Cdt to induce dysplasia and carcinogenesis in some murine models. Ge and coworkers reported that bacteria producing Cdt are able to enhance the carcinogenic potential in different mice models at least in part via the elevation of DSBs and increased activation of the STAT3 signaling pathway [32,54,55]. Moreover, in human CRC patients, Cdt-producing bacteria were found close to tumors but not in the healthy part of the colon [56]. Even if Cdts have shown to possess carcinogenic properties, they may possibly be exerted only under specific circumstances, such as inflammatory conditions and genetic susceptibility to cancer.

### 2.2. Colibactin

In addition to the Cdts, the DNA interacting colibactin has also been associated with the formation of DSBs and the introduction of genomic instability (Figure 3B). Colibactin is a genotoxin secreted by *Escherichia coli* strains of the phylogenetic group B2. These organisms harbor a 54-kb biosynthetic gene cluster of 19 genes, named clbA to clbS (also referred to as pks), that encodes a non-ribosomal peptide synthetase-polyketide synthase (pks) assembly line, which has been implicated in colibactin biosynthesis [57]. It is worth noting that, although colibactin is not a “canonical” protein toxin, it is constantly included in all scientific writings dealing with bacterial toxins and cancer. *E. coli* containing the pks island (pks+ *E. coli*) frequently colonize the newborn gut in humans and are present in the microbiota of inflammatory bowel disease and CRC patients [56]. Pks island is also present in the probiotic *E. coli* Nissle 1917 [58,59] and in other members of the Enterobacteriaceae family, such as *Klebsiella pneumoniae*, *Enterobacter aerogenes*, and *Citrobacter koseri* isolates [60], all encoding the biosynthesis of a non-proteogenic metabolite [61]. Colibactin’s chemical structure and the molecular mechanism underlying its genotoxic effects have long remained unknown since colibactin is unstable and is produced in vanishingly small quantities. Recent studies suggest that colibactins are unsaturated imines, that are potent DNA damaging agents [62], allowing researchers to probe for causal relationships between the metabolite and inflammation-associated CRC [63]. The products of pks island have shown to exert many different functions, highlighting the complexity of this region: Genotoxic, antimicrobial, and analgesic activities [64,65,66]. Similarly to mechanisms of Cdts genotoxicity, colibactin secreted by pks+ *E. coli* promote DDRs by inducing DSBs, probably by DNA alkylation on adenine residues [63,67], SSBs and inter-strand crosslinks in DNA of eukaryotic cells [57,68]. These mechanisms affect cell cycle leading to cell death by apoptosis or senescence and/or to DNA repair [69]. As a side effect of their mode of action, colibactin-producing bacteria also induce incomplete DNA repair, chromosomal instability, and anchorage-dependent colony formation, phenotypes that can promote cancer formation. Pks+ *E. coli*, more often isolated from tissue of CRC patients compared to healthy individuals, induce in vitro a cytopathic response in primary colon epithelial and CRC cell lines, suggesting that these isolated strains may be involved in the initiation and development of CRC [70]. Moreover, the same mutational signature detected in a subset of human cancer genomes was found in a human intestinal organoid affected by prolonged pks+ *E. coli* exposition [71]. Several studies show that colibactin-producing *E. coli* bacteria (CoPEC) are more common in individuals with familial adenomatous polyposis (FAP) and CRC, than in cancer-free individuals [56,72]. Pks+ *E. coli* infection has also shown to induce cellular senescence characterized by the production of growth factors that promote the proliferation of uninfected cells and, subsequently, tumor growth in a xenograft mouse model [73]. Increasing evidence show the role of immune environment, especially of antitumor T-cells, in CRC development. The colonization of CRC patients by CoPEC is associated with a decrease of tumor-infiltrating T lymphocytes (CD3+ T-cells). Similarly, in mice, CoPEC chronic infection decreases CD3+ and CD8+ T-cells and increases colonic inflammation, suggesting that pks+ *E. coli* could promote a pro-carcinogenic immune environment through impairment of antitumor T-cell response [74]. Moreover, colitis-susceptible IL-10-deficient mice showed increased formation of invasive carcinoma when colonized with CoPEC, whereas deletion of the pks genotoxic island from these *E. coli* strains decreased tumor multiplicity and invasion [72,75]. Despite the evidence that colibactin-producing *E. coli* bacteria can induce CRC, the relationship between CoPEC and the development of CRC must be further investigated to clarify if the presence of CoPEC in the cancerous lesion can be an effect rather than a cause of carcinogenic process [76].

## 3. Bacterial Protein Toxins Causing Cell Signaling Alterations

Certain bacterial toxins, such as *Bacteroides fragilis* toxin and Cytotoxic Necrotizing Factor 1, can contribute to CRC development or progression through alteration of crucial cell signaling pathways involved in cell proliferation and cell death.

### 3.1. Bacteroides Fragilis Toxin (BFT)

Enterotoxigenic *Bacteroides fragilis* (ETBF) is a human colonic commensal. A subset of strains can produce a proteolytic enterotoxin, named *B. fragilis* toxin (BFT), or fragilysin, that causes secretory diarrhea and colonic epithelial damage [77]. BFT is among the most studied virulence factors of *B. fragilis*, and current evidence suggests that this toxin may be a driver for chronic colitis and CRC [78,79,80] (Figure 3C).

BFT is a secreted 20 kDa zinc-dependent metalloprotease that binds to an unidentified intestinal epithelial cell receptor and causes the cleavage of the extracellular domain of the tumor suppressor protein E-cadherin [81,82,83], thus inducing loss of cell–cell contacts, cell rounding, and cell proliferation [84]. The intracellular domain of E-cadherin is normally bound to α and β-catenin [85]. When β-catenin is dissociated from E-cadherin, it can function as a transcription factor in a Wnt-dependent manner, inducing cell proliferation through activation of the c-Myc pathway [85]. BFT-mediated cleavage of E-cadherin promotes the migration of β-catenin to the nucleus [86]. Also, E-cadherin cleavage by BFT triggers the induction of MAPKs and the NF-κB pathway, thus increasing the secretion of IL-8, a chemokine that attracts polymorphonuclear cells. NF-κB activation controls fluid secretion of intestinal cells through induction of COX-2 and an increase in prostaglandin E2 levels. COX2 and heme oxygenase-1 induction by BFT is related to a delay of apoptosis in intestinal epithelial cells [87,88]. Moreover, it has been recently demonstrated that signaling pathways affected by the toxin cause differential gene expression and epigenetic changes in HT29 cells [89].

The relevance of the above findings in cell cultures has been elaborated in animal models of ETBF-associated disease, where ETBF has been shown to contribute to colon carcinogenesis. Rabizadeh and coworkers and Rhee and coworkers showed, for the first time in specific pathogen-free C57BL/6 mice, that ETBF causes acute and persistent colitis in mice, driven by cleavage of E-cadherin in vivo, activation of STAT3, c-myc expression and concomitant cell proliferation [90,91]. Chronic colitis, observed between 7 days and up to 16 months after infection, shows progressive hyperplasia of the colonic crypts in accordance with BFT-dependent induction of a cell hyperproliferative program [90,92]. Tumor-prone mice co-colonized with *E. coli* expressing colibactin and ETBF showed increased IL-17 in the colon and DNA damage in colonic epithelium, with faster tumor onset and greater mortality compared to mice with either bacterial strain alone, suggesting an unexpected link between early CRC and tumorigenic bacteria [79,80,93,94].

The first study demonstrating an increased prevalence of ETBF in the stool specimens of CRC patients (38%) compared with the control group (12%) was conducted by Ulger Toprak and coworkers [95]. Different reports have shown an association between *bft* gene and CRC, particularly in the late stage (III/IV) of CRC [96,97,98,99] and significant associations of ETBF with tubular adenomas, serrated lesions, and low-grade dysplasia [100]. Interestingly, patchy bacterial biofilms composed predominately of *E. coli* and *B. fragilis* were identified in the colonic mucosa of patients with FAP, who develop benign precursor lesions (polyps) early in life. Genes for colibactin (*clb*) and *B. fragilis* toxin (*bft*) were highly enriched in FAP patients’ colonic mucosa compared to healthy individuals [90]. In a very recent study conducted in Iran, where CRC is one the most common cancers, the frequency and abundance of ETBF in biopsy samples of patients with CRC and precancerous conditions were compared to those of the individuals with no personal or familiar history of colorectal disease. The results obtained showed an increased positivity of ETBF in patients with precancerous and cancerous lesions and suggested that mucosal BFT exposure is common and could be a risk factor and a screening marker for developing CRC [101].

### 3.2. Cytotoxic Necrotizing Factor 1 (CNF1)

The cytotoxic necrotizing factor 1 (CNF1) is a protein toxin produced by certain pathogenic *E. coli* strains that permanently activates the Rho GTPase family proteins Rho, Rac, and Cdc42 [102], pivotal molecular switches that oscillate between a GDP-linked inactive form and a GTP-linked active form. CNF1 activates Rho GTPases through deamidation of a glutamine residue located in the switch two domain, involved in GTP hydrolysis (glutamine 63 in Rho) [103,104] or 61 in Cdc42 and Rac [105]. Rho, Rac, and Cdc42 primarily regulate the actin cytoskeleton organization [106], but they are also involved in several crucial cellular processes including regulation of transcription, cell migration, cell polarity and cell cycle progression [107] (Figure 3D).

Due to the large spectrum of Rho GTPases activities, their activation induced by CNF1 generates different and unexpected abilities in epithelial cells that suggest a reprogramming of cells. Being Rho GTPases the master regulators of the actin cytoskeleton, CNF1 leads to a number of actin-dependent events in cells, such as micropinocytosis [108], increase in cellular motility [109], and multinucleation [110], the last one resulting from nuclear constriction, unsuccessful cytodieresis and budding or multipolar metaphases [111]. CNF1 is classified as cyclomodulin due to its role in the perturbation of host cell cycle [112,113]. In fact, it prevents cell cycle progression and arrest cells in G2/M phase, stimulates DNA synthesis and promotes the transition of quiescent cells into proliferation [114,115,116]. An interesting work reported that CNF1 treatment blocks mitosis/cytokinesis and elicits endoreplication and polyploidization in in vitro CRC cells [117]. CNF1-treated cells undergo reversible senescence and depolyploidization is described as a survival route. It is interesting to note that Zhang and coworkers (2018) [117] described that CNF1 treatment increases the incidence of chromosome aneuploidy and enhances micronuclei formation, both phenomena being a clear sign of genomic instability [117,118]. CNF1 also triggers events not directly linked to the actin cytoskeleton. It has been reported, in fact, that CNF1 stimulates NF-κB [115,119,120] via the Akt/IκB kinase pathway, thus favoring cell survival. Interestingly, CNF1 is able to induce an enrichment of the mitochondrial network and increase of the ATP production in cells [121]. Actually, CNF1 protects from apoptotic stimuli by increasing the amount of anti-apoptotic proteins and by generating a Rho-dependent cell spreading [122,123]. Last but not least, CNF1 induces the production of pro-inflammatory cytokines, of COX-2 and stimulates cells to enter the cell cycle [124]. It is interesting to note that all the effects generated by CNF1, herein described, are reminiscent of transformed cells [125]. In this context, Guo and coworkers recently reported that CNF1 secreted by uropathogenic *E. coli* strains accelerates prostate cancer progression [126]. Their studies demonstrate that CNF1 promotes pro-migratory and pro-invasive activity through Cdc42 activation, PAK1 phosphorylation and up-regulation of MMP-9 expression. In a xenograft mouse model, CNF1 promoted pulmonary metastasis through the same mechanism. Very recently, we demonstrated that CNF1 is able to induce epithelial-to-mesenchymal (EMT) transition in intestinal epithelial cells. In fact, CNF1 speeds up, in in vitro systems, the heal of the wound and induces the expression of EMT-related transcription factors together with the activation of the mTor pathway [127]. However, it is worth noting that the ability of CNF1 to promote carcinogenic traits is critically dependent on inflammation and on the cell type. In fact, whereas already transformed cells undergo EMT after CNF1 exposure, normal cells require the presence of an inflammatory environment in order to go through EMT [127].

Intriguingly, the gene coding for CNF1 has been associated with the intestinal mucosa of patients with Crohn’s disease [128]. More strikingly, the presence of colonic mucosa-associated *E. coli* in biopsies from patients with CRC or diverticulosis indicates that CNF1-producing *E. coli* strains colonize more frequently CRC than diverticulosis samples [56].

## 4. Other Bacterial Factors That May Influence Host Cell Transformation

Bacteria can promote host cell transformation also via protein virulence factors that are not properly protein toxins [52]. These are bacterial effector proteins, such as the Avirulence protein A or cell surface components, such as the *Fusobacterium nucleatum* FadA. Only these two factors will be herein described as examples.

### 4.1. Avirulence Protein A (AvrA)

*Salmonella* infection, in addition to the acute enteric Salmonellosis, can become chronic and increase the risk of other gastrointestinal diseases, including chronic inflammation and cancer. It is well-known that long-standing *Salmonella* infection increases the risk of gallbladder cancer [129,130]. Avirulence protein A (AvrA) is a bacterial effector protein produced by *Salmonella* strains that is crucially involved in *Salmonella*-induced intestinal inflammation and chronic infection in vivo [131]. AvrA is a 33 kDa multifunctional protein able to influence eukaryotic cellular pathways by regulating ubiquitination and acetylation of target proteins [131,132,133], with the final results of modulating inflammation, proliferation, and apoptosis [132,134,135,136] (Figure 3E). In 2014, Lu and coworkers indicated AvrA as a clear promoter of CRC development in vivo [137]. They examined the effects of chronic infection with AvrA-expressing *Salmonella* and demonstrated that AvrA enhances proliferation and promotes colonic tumorigenesis and tumor progression. This is achieved by the induction of β-catenin and STAT-3 signals, that co-regulate in a positive feedback loop, AvrA chronically activating β-catenin [133] and persistently upregulating STAT-3 [138]. Activation of β-catenin, STAT-3, and downstream effectors may be a critical point in the initiation and the progression of CRC. Recently, Lu and coworkers demonstrated the presence of *Salmonella* AvrA protein in colorectal mucosa derived from experimentally infected mice as well as in human clinical specimens [139]. Additionally, they were able to detect anti-AvrA antibodies in mouse serum after *Salmonella* infection.

It is worth noting that *S. typhi* also produces Cdt that, as above reported (see Cdt section), possesses a carcinogenic potential. To our knowledge, no studies so far exist that correlate the activity of Cdt and AvrA in tumor development, although we cannot rule out cooperation or a synergism between the two toxins.

### 4.2. Fusobacterium Nucleatum Adhesin A (FadA)

*Fusobacterium nucleatum* is an obligate anaerobe Gram-negative bacterium that represents an important component of the oral microbiota as well as of the upper respiratory tract, the gastrointestinal tract, and the genitals [140]. *F. nucleatum* is coming out as a potential candidate for CRC susceptibility and, interestingly, has been discovered to be enriched in the carcinomas of CRC patients [141,142]. Noteworthy, its enrichment in cancer tissue is associated with shorter survival and its expansion, usually associated with periodontal disease, is linked to an increased CRC risk [143]. Altered levels of *F. nucleatum* were also found in the different stages of CRC development [144,145,146].

Mechanisms underlying *F. nucleatum*’s contribution to CRC development comprise immune modulation, miRNAs, bacteria metabolism and virulence factors, as already reviewed [147,148]. The *Fusobacterium* adhesin A (FadA), a bacterial cell surface adhesion component, is a protein virulence factor identified from *F. nucleatum* [149,150] that seems to be responsible for the carcinogenic effect of *F. nucleatum*. FadA binds host E-cadherin thus mediating attachment and invasion into cells. It modulates E-cadherin-mediated cellular signaling, in turn, activating β-catenin and leading to the expression of transcription factors, oncogenes, *Wnt* genes and inflammatory genes, and promoting CRC cells proliferation [146] (Figure 3F). Very recently, Rubinstein and coworkers reported that FadA modulates Wnt/β-catenin signaling in cancer cells via the up-regulation of Annexin A1 expression, a critical growth stimulator of CRC [151]. Stimulation of CRC occurs through an E-cadherin-dependent, positive feedback loop of FadA and Annexin A1, this last being uniquely present in the cancerous cells. In this context, *F. nucleatum* can induce EMT and FadA seems to play a major role in this process [152].

In xenograft mice, FadA promotes inflammation and E-cadherin-mediated CRC tumor growth. Interestingly, tissues from human adenomas and adenocarcinomas have elevated FadA gene expression levels. In particular, gene levels in the colon tissue from patients with adenomas and adenocarcinomas are > 10–100-times higher compared to normal individuals and the increased FadA expression in CRC correlates with an increase in the expression of inflammatory and oncogenic genes [146].

## 5. Bacterial Protein Toxins in Cancer Therapy

It is well established that toxigenesis, the capacity to produce toxins, is one of the principal mechanisms by which many bacterial pathogens produce disease. However, although several bacteria and their toxins are indicated as cancer-causing agents, recent research reveals intriguing results that suggest their potential use in cancer therapy, a Janus aspect that will be herein briefly presented. As evidenced in the previous sections, bacterial toxins are extremely effective enzymes, with high specificity towards their cellular substrates, often represented by molecules playing a key role in the host cellular signaling pathways. Few toxin molecules are sufficient to change the cellular morphology and function or even kill a cell. Thus, the most applied idea is to combine the specificity of a ligand towards a peculiar surface receptor expressed on cancer cells with the toxin catalytic portion for killing tumor cells. These molecules are generally composed of a receptor-binding moiety, generally an antibody (giving rise to an immunotoxin, IT), and a second part, represented by the catalytic moiety of a toxin, responsible for the toxin-induced lethality [153]. To date, although hundreds of different IT constructs have been developed against a number of malignancies, and numerous clinical trials of ITs have been conducted or are under way, only three ITs have been approved for human use thus far [154,155] and they have shown better results in hematological cancer treatment with respect to solid tumors.

However, the field of bacterial toxins offers a mine of resources and a number of bacterial toxins are under study as therapeutic tools for different types of cancer, some of them displaying useful characteristics to specifically kill cancer cells of various origin, including CRC [156,157,158]. Very recently, the bacterial cancer therapy is emerging as a promising alternative anticancer approach on tumor cells also in vivo, bacterial strains possessing the ability to directly target hypoxic regions of tumors and secrete therapeutic molecules, thus leading to cancer cell death [155]. Finally, the manipulation of gut bacteria for therapeutic purposes could include strategies against pks+ *E. coli* strains in humans since the causal link to CRC is very close to being established [71].

## 6. Conclusions

Although the role of bacterial protein toxins in the CRC onset and progression is still under debate, an exciting new finding strongly supports this view. It has just been discovered, in fact, that mutations detected in CRC match the gut bacterium pks+ *E. coli* signature [71]. This study evidences the potential contribution of colibactin to the onset of cancer in humans, and also supports the fact that the gut bacteria community may contain strains with pro-carcinogenic properties. The bacterial toxins that induce hallmarks of cancer enroll two main routes, one leading to a direct attack to DNA, as in the case of colibactin or CDT that cause mutations and genome instability, the other engaging signaling pathways that modulate cell proliferation, replication, and death, ultimately resulting in transformation, as for BFT, CNF1, FadA, or AvrA (Table 1).

Also, several bacterial toxins can control tumor-promoting inflammation. The secretion of protein toxins is one of the main strategies employed by intestinal bacteria to manipulate target cells. These toxins are expressed and secreted through specialized secretion systems for transport across bacterial outer membranes. However, the release of protein factors from internalized bacteria or the direct interaction of the target cell with bacterial surface moieties are efficient systems to interact with the target cell as well. Bacterial toxins are among the oldest molecules that evolved as a virulence factor for the bacteria and, therefore, studying their evolutionary findings can contribute to explain their origin, the rationale underlying their diversity, and the purpose of their production [159]. Such virulence factors clearly provide a selective advantage to the bacteria and, importantly, add an extra energy burden for the bacterial cells and also divide labor between bacterial cells, with only a fraction of them that are toxigenic [160]. In general, to control the host cell machinery, bacterial protein virulence factors manipulate host cell signaling pathways and affect host cell integrity, which can coincidentally induce cellular malignancies. Hence, each toxin is potentially offensive for the host and defensive for the producing bacterium and, also, can possibly contribute to a bacterial behavior completely unrelated to its pathogenicity, such as the pro-carcinogenic activity. Furthermore, these bacterial protein factors can also provide benefits to the bacteria not directly connected to an interaction with their host, such as favoring biofilm formation, bacterial motility, and niche establishment [161]. On the other hand, the emerging statement that bacteria and their products can be involved in cancer promotion does not explain if and eventually how such a carcinogenic activity would be beneficial for the pathogens. Bacteria normally produce virulence factors to suit their own needs, including the protection against the immune system or the capacity to enter and invade the target cells. Sophisticated examples are furnished by *Salmonella* effector proteins, which in tandem induce the entrance and subsequently the permanence of the bacterium inside the cells by modulating the actin cytoskeleton activity [162,163,164]. In contrast, bacterially-induced cancer is most probably a side effect, an unwanted consequence of the bacterial infection also because cancer usually occurs long after the bacterium and its effectors have left the host.

As concerns the gut microbiota-mediated carcinogenesis, as already stated in the Introduction, such a complex phenomenon relies upon several factors, principally including the microbiota composition, the metabolization of dietary components, and the immune system. A balanced intestinal microbiota confers resistance to the colonization of pathogenic bacteria by employing different strategies, such as competing both for nutrients and for the attachment to the colonic epithelial cells; by producing and secreting compounds with antimicrobial properties; by reinforcing the epithelial tight junctions [165]. In this context, gut commensals dysbiosis can play a pivotal role. In fact, some bacterial subpopulations can rise thus creating an unfavorable microenvironment in term of inflammation and release of pro-carcinogenic factors. These phenomena may favor the consequent development of pathogenic populations that can negatively affect the host’s gut and metabolism as well as the immune system functionalities, thereby triggering tumor growth [166,167]. Several studies have evidenced that about 15–16% of human cancers are triggered by bacteria [168,169], a concept that is strongly supported by the breakthrough that many gastric cancers can be attributed to *H. pylori* infection [170]. As concerns the CRC, a meta-transcriptomic analysis has revealed a high expression of pro-carcinogenic toxins in human CRC tissue samples although toxin-producing bacteria constitute only a minor part of the colonic microbiota [171]. Furthermore, prospective and case-control studies show that the gut microbiota of patients with CRC usually contains a high number of pro-inflammatory and toxigenic bacteria as well as bacteria that produce carcinogenic metabolites. Hence, alterations in the gut microbiota composition are now considered as a risk factor for the development of CRC, and specific pathogens apparently contribute to causation and disease progression. The etiology of CRC, however, is just rarely attributable to the presence and activities of single pathogenic species [172] and the contribution of protein toxins as well as of other effector proteins is normally effective in causing cancer only in the concomitant presence of other factors, such as the cumulative effects of microbial metabolites, pre-existing mutations or an inflammatory environment. A proinflammatory state, which can be triggered by alteration of the intestinal microbiota, could compromise the mucosal barrier integrity, thus possibly inducing a widespread and even systemic inflammation [173]. This last finding is important since it indicates that the inflammation eventually raised in the intestine, where is the major concentration of resident bacteria, can also affect distant body districts [174]. In this context, it is interesting to note that the majority of pro-carcinogenic toxins herein described are produced by *E. coli* or Gram-negative bacteria that are able to release lipopolysaccharide, also in form of extracellular vesicles. Such bacterial product can enter the systemic circulation and elicit a variety of immunological and metabolic responses [175,176]. Inflammation plays an important role in CRC [177] and it seems to be crucial in the toxin-induced pro-carcinogenic activity (see Figure 3). The establishment of chronic inflammation, in particular, can crucially contribute to cancer via different mechanisms, including the induction of the EMT, a process involved in metastasis, invasion, and progression of various cancers [178,179]. The ability to induce factors involved in EMT has first been reported for the protein toxins CagA and CagE, from the pro-carcinogenic bacteria *H. pylori* whose presence increases the proneness of patients to gastric cancer [180,181,182]. More recently, the protein toxin CNF1 from *E. coli* has been proved to trigger EMT only in already transformed epithelial intestinal cells, whereas it requires the presence of an inflammatory environment in non-transformed cells [127]. This suggests the crucial role of inflammation in the CNF1-producing *E. coli* pro-carcinogenic activity in the gut. Also, the surface adhesin FadA appears to be crucial in the EMT induced by *F. nucleatum*, a bacterium that is enriched in CRC tissue [183]. It is interesting to note that, although interacting with host cells in different ways, diverse protein virulence factors may reach the same outcome, such as for example the induction of EMT, the activation of β-catenin, or uncontrolled cell proliferation.

Finally, even if further studies in humans and experimental animal models are necessary to definitively prove the pro-carcinogenic activity of certain bacterial protein toxins, the actual knowledge on these factors paves the way to their inclusion in cancer screening programs as tools that may predict the risk of CRC.

## Figures and Tables

**Table 1:** Bacterial virulence factors indicated as CRC promoters.

Bacterium	Type of Virulence Factor	Virulence Factor	Activity and Pro-Carcinogenic Outcomes	References
*Gram-negative bacteria*	Protein toxin	Cytolethal distending toxin (Cdt)	DNA damage, cell cycle arrest, survival, mitogen-activated protein kinase (MAPK) and STAT3 pathways, transformation	[24,25,26,27,28,29,30,31,32,33,34,35,36,37,38,39,40,41,42,43,44,45,46,47,48,49,50,51,52,53,54,55]
*Escherichia coli*	Protein toxin	Colibactin	DNA damage, apoptosis and senescence, proliferation	[57,58,59,60,61,62,63,64,65,66,67,68,69,70,71,72,73,74,75,76]
*Bacteroides fragilis*	Protein toxin	*B. fragilis* toxin (BFT)	Cell proliferation, activation of MAPK and NF-κB pathway and of COX2, delay of apoptosis	[77,78,79,80,81,82,83,84,85,86,87,88,89,90,91,92,93,94,95,96,97,98,99,100,101]
*Escherichia coli*	Protein toxin	Cytotoxic necrotizing factor 1 (CNF1)	Activation of Rho GTPases, transformation, NF-κB activation and survival, EMT	[56,102,103,104,105,106,107,108,109,110,111,112,113,114,115,116,117,118,119,120,121,122,123,124,125,126,127,128]
*Salmonella* sp.	Effector protein	Avirulence protein A (AvrA)	β-catenin and STAT-3 induction, proliferation and colonic tumorigenesis	[52,129,130,131,132,133,134,135,136,137,138,139]
*Fusobacterium nucleatum*	Cell surface adhesion protein	*F. nucleatum* Adhesin A (FadA)	Wnt/β-catenin activation, inflammatory genes induction and CRC cells proliferation, EMT	[52,140,141,142,143,144,145,146,147,148,149,150,151,152]

## Abbreviations

ATM	ATM-and Rad3 related
APC	Adenomatous polyposis coli
AvrA	Avirulence protein A
BFT	*Bacteroides fragilis* toxin
CNF1	Cytotoxic necrotizing factor 1
CoPEC	Colibactin-producing *Escherichia coli* bacteria
CTCL	Cutaneous T-cell lymphoma
DT	Diphtheria toxin
EMT	Epithelial-to-mesenchymal
ETBF	Enterotoxigenic *Bacteroides fragilis*
FadA	*Fusobacterium nucleatum* adhesin A
FAP	Familial adenomatous polyposis
HCECs	Human colon epithelial cell lines
IT	Immunotoxin
KRAS	Kirsten-ras
MAPKs	Mitogen-activated protein kinases
SE	Staphylococcal enterotoxin
SEA	Staphylococcal enterotoxin A
STAT3	Signal transducer and activator of transcription 3
